# Picture quiz: non-optical measures to support children with low vision

**Published:** 2012

Throughout this issue of the journal, there are practical suggestions for supporting people with low vision by improving their environment. However, each person and each situation is different. Look at these pictures, read the questions, and try your best to come up with as many ideas as possible. Answers given below.

**Figure:**
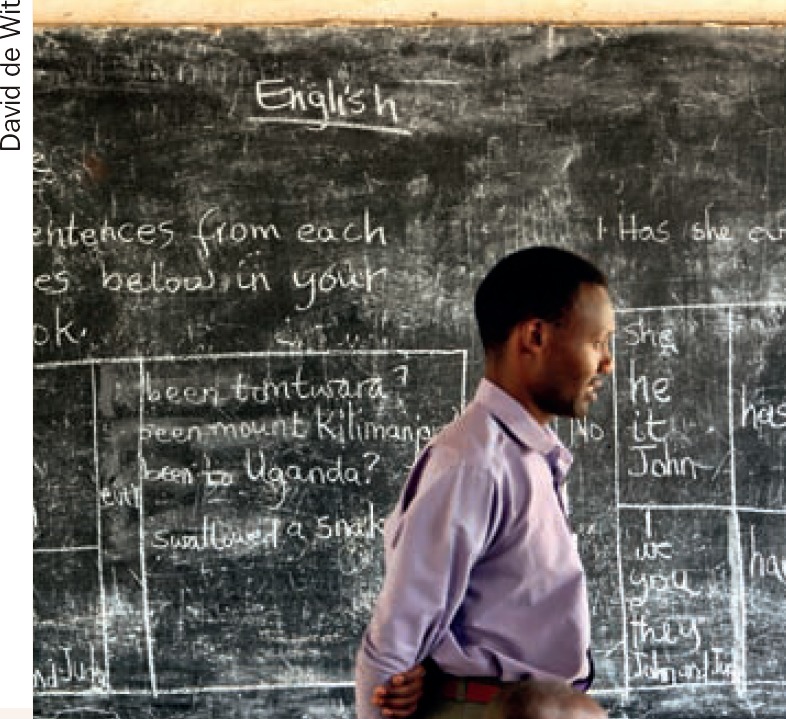
1 If there is no telescope, how can you ensure a child with low vision can access the information on the blackboard?

**Figure:**
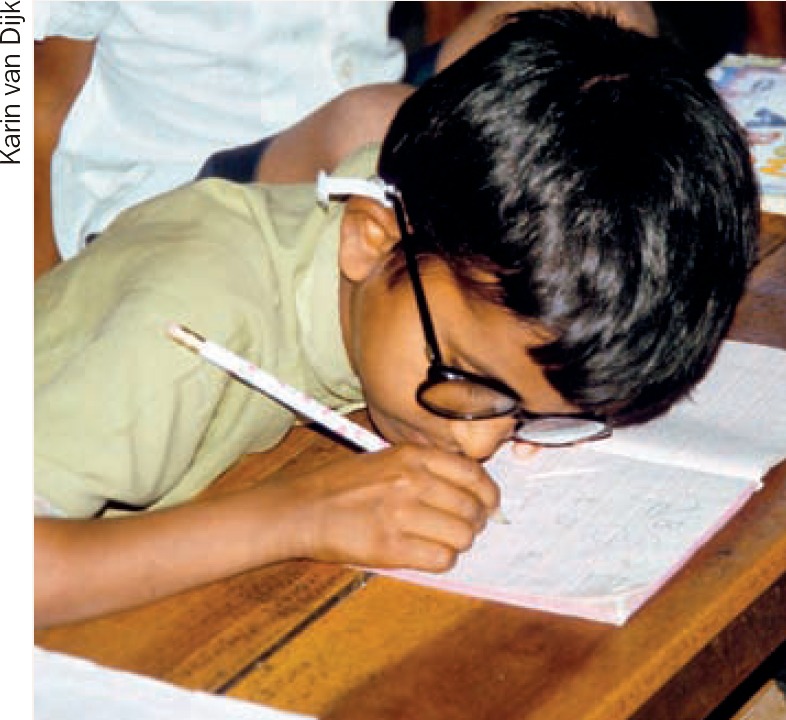
2 How can you increase legibility and comfort for this student who is writing notes?

**Figure:**
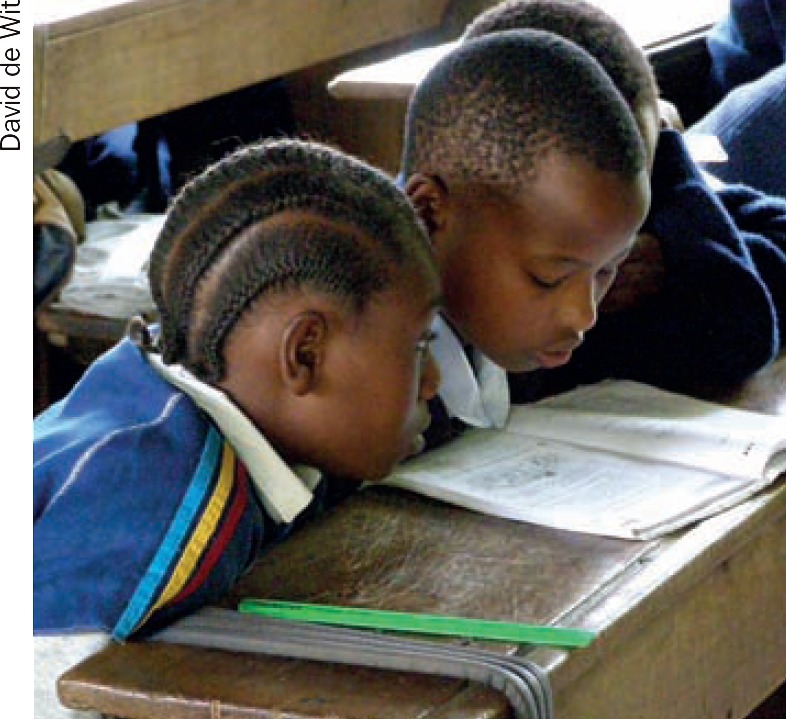
3 How can you ensure the child with low vision can read the textbook?

**Figure:**
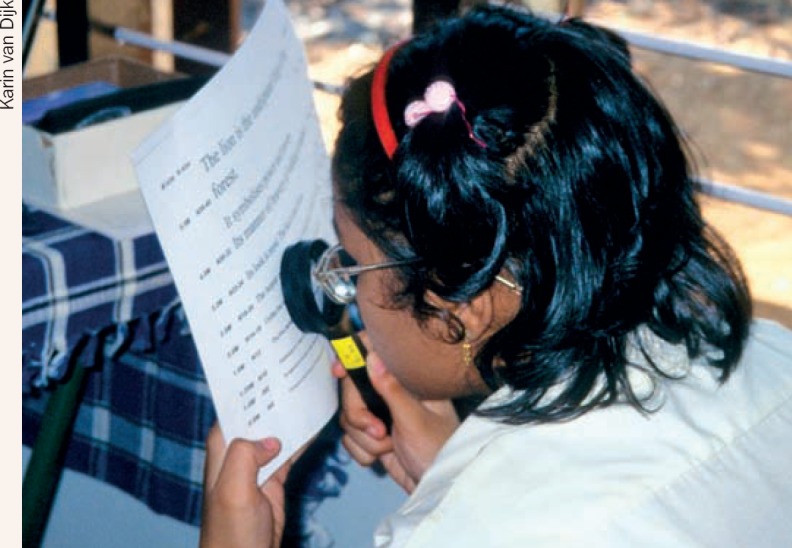
4 What advice can you give to improve reading?

**Figure:**
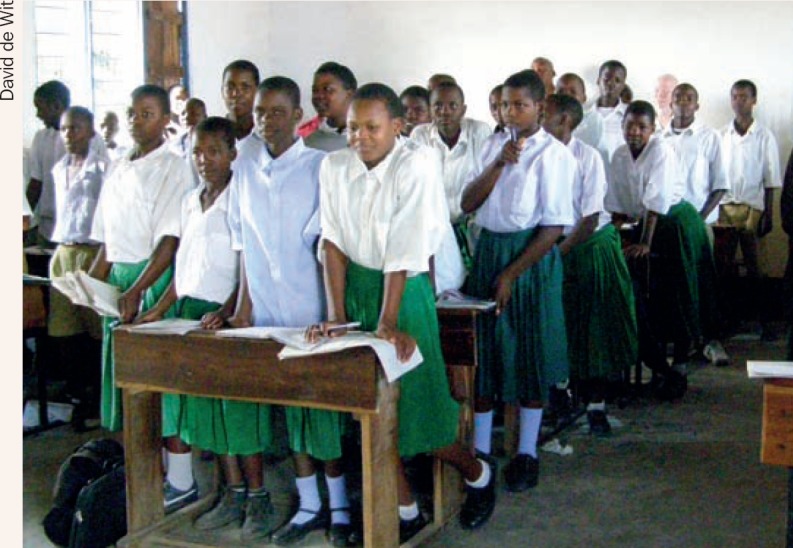
5 Find the child with albinism. Is this the best place for him? Please give advice.

## ANSWERS

**Question 1**

- The teacher can place the child with low vision as close to the blackboard as possible
- The teacher can ensure good contrast on the blackboard by cleaning it regularly, writing in a larger size, where possible, and organising the text clearly
- The teacher can say what she writes while she is writing it
- The child with low vision can copy from her friend's notebook
- A friend can say the words on the board so the child with low vision can hear and write them down.

**Question 2**

- The child can write with a very dark pencil or black pen (preferred)
- The child can be encouraged to write in a size that the child himself can easily see, at a better distance. Consider providing a bigger notebook
- Use a stand for writing – this brings the book closer and is more comfortable.

**Question 3**

- Make sure the child with low vision has her own textbook
- Put the book on a reading stand.

**Question 4**

- Use a reading stand and lay the text flat on the stand
- If no reading stand is available, use a clipboard or piece of board to keep the text flat
- Use the hand magnifier at a distance where the text is as large as possible and still clear. At the moment, the magnifier is too close to text to give enough magnification.

**Question 5**

- Children with albinism should not sit in a place where direct sunlight falls on them
- Place the child nearest the blackboard.

